# Supplementary material for: Syntactic processing engages the semantic control network
Source: Imaging Neurosci (Camb). 2026 Jun 22;4:IMAG.a.1281. doi: 10.1162/IMAG.a.1281 (PMC13288499; doi:10.1162/IMAG.a.1281)
Supplement: Supplementary Material [file IMAG.a.1281_supp.pdf]

Supplementary Table 1. The 66 experiments included in the meta-analysis of syntactic processing

Study	DOI	N	Modality	Syntactic manipulation	Space	Contrast	X	Y	Z
Bahlmann et al. (2007)	10.1002/hbm.20318	12	visual	complexity	MNI	non-canonical > canonical word order (subject first > object first)	-40	-52	28
							-44	24	28
Ben-Shachar et al. (2003)	10.1111/1467-9280.01459	11	auditory	complexity	TAL	transformational > non-transformational sentence	-47	18	7
							-37	-40	20
Bornkessel-Schlesewsky et al. (2009)	10.1016/j.bandl.2009.09.004	28	visual	complexity	TAL	object-subject > subject-object sentence	-53	11	5
							-34	23	5
							-40	5	32
							31	20	6
							-7	23	41
							-32	-58	41
							-14	1	18
Caplan et al. (1998)	10.1006/nimg.1998.0412	16	auditory	complexity	TAL	cleft object > cleft subject sentence	-52	18	24
Caplan 1998	10.1162/089892998562843	8	visual	complexity	TAL	center-embedded > right-branching sentence	10	6	52
							-2	6	40

							-42	18	24
Caplan 2000	10.1002/(SICI)1097-0193(200002)9:2<65::AID-HBM1>3.0.CO;2-4	11	visual	complexity	TAL	Center-embedded > right-branching clauses	-46	36	4
							-14	-20	4
							-10	-36	40
							0	56	8
Caplan et al.. (2008)	10.1162/jocn.2008.20044	16	visual	complexity	MNI	object-extracted relative clause > subject-extracted relative clause (plausible unconstrained sentences)	-52	-44	-2
							-46	-56	16
							-62	-18	-1
							-38	30	0
							-44	34	-14
							-46	24	-8
							-54	18	28
							-46	14	18
						object-extracted relative clause > subject-extracted relative clause (plausible constrained sentences)	-24	-76	32
							-28	-84	22
							-24	-86	32
							-58	-46	2
							-50	-60	12

							-50	-42	-4
Carreiras 2015	10.1016/j.neuroimage.2015.06.075	32	visual	violation	MNI	number disagreement > agreement (subject–verb and determiner–noun)	32	26	-2
							28	20	4
							-36	-42	42
							-28	24	0
							-50	10	26
							-42	20	-4
							-40	14	28
							-46	-2	48
							-6	4	62
Chen 2006	10.1016/S0010-9452(08)70397-6	12	visual	complexity	TAL	Object relative > subject relative clause	-60	16	14
							-46	-68	30
							-41	-43	40
							-53	9	40
							36	-61	38
							25	-56	48
Constable et al. (2004)	10.1016/j.neuroimage.2004.01.001	20	visual & auditory	complexity	TAL	object relative > subject relative sentence	-51	-58	3

							-49	11	13
							-36	-64	31
							-36	4	46
							-2	6	33
							-3	-24	15
							44	6	2
							43	14	23
Cooke et al. (2001)	10.1002/hbm.10006	7	visual	complexity	TAL	object- > subject-relative center-embedded clause (short antecedent-gap distance)	-48	-68	-8
							-4	-92	-8
							28	-68	-20
						object- > subject-relative center-embedded clause (long antecedent-gap distance)	-40	-76	-4
							-32	-20	-20
							36	-40	-12
							16	-92	-12
Cooke et al. (2006)	10.1016/j.bandl.2005.07.072	15	visual	violation	TAL	Inflectional morphology violation > correct sentence (early phase)	-60	8	24
							-60	-52	8
							4	16	48
						Grammatical category violation > correct sentence (early phase)	-44	12	24

							-60	-52	4
							-12	8	52
							48	8	32
							36	-64	44
						Grammatical category violation > correct sentence (late phase)	-48	12	0
							-44	-52	0
						Transitivity violation > correct sentence (early phase)	-52	0	40
							4	8	52
							48	8	40
						Transitivity violation > correct sentence (late phase)	-48	8	12
Feng et al. (2015)	10.1016/j.jneuroling.2014.09.002	18	visual	complexity	MNI	Passive > active sentences	-48	26	0
							-56	-44	12
							-44	-2	46
							-62	0	-14
							-18	-48	2
							-34	-22	24
							32	0	20
							24	-28	-26

Fiebach et al. (2005)	10.1002/hbm.20070	14	visual	complexity	TAL	object questions with long > short dislocation	-44	21	11
							-45	21	10
							-46	17	4
							-54	7	28
							-54	-27	-1
							-52	-46	6
							45	-18	-3
							-18	-18	12
Friederici et al. (2006)	10.1093/cercor/bhj106	13	visual	violation	TAL	grammatically incorrect > correct sentence	-43	-23	52
							46	-43	52
							34	-65	44
							-31	-70	-17
Friederici et al. (2010)	10.1002/hbm.20878	17	auditory	violation	MNI	grammatically incorrect > correct sentence	-54	-16	4
							-60	-42	6
							-62	18	16
							-12	-28	2
							44	-20	6
Grewe et al. (2005)	10.1002/hbm.20154	16	visual	complexity	TAL	permuted non-pronominal (N-OS) > non-permuted non-pronominal (N-SO)	-32	20	3
							-52	14	15
							-2	32	30

							-38	8	38
							44	26	18
							38	20	6
							46	11	9
Grewe 2007	10.1016/j.neuroimage.2006.11.045	19	visual	complexity	TAL	object > subject-initial sentences	-53	10	15
							-20	-86	24
							28	-8	15
Herrmann et al. (2012)	10.1002/hbm.21235	25	auditory	violation	MNI	syntactically incorrect > correct	-54	8	10
							-60	-22	-2
							-54	5	-14
							57	-28	1
							60	-4	-8
							-45	-22	1
							48	-22	7
Husband et al. (2011)	10.1162/jocn_a_00040	19	visual	violation	TAL	ungrammatical > grammatical sentence	-45	-65	30
							-46	1	-27
							-29	-39	57
							-6	-15	50
							-45	-65	29
							-6	58	23
							45	-65	32
							45	-65	28
Iwabuchi	10.1016/j.jneuroling.2020.100893	23	visual	complexity	MNI	scrambled subject-second >	-3	17	55

2020						canonical subject-first sentence			
							33	23	-8
							-42	14	25
							-30	26	-2
							-39	2	43
							-30	-61	43
Kambara et al. (2013)	10.1016/j.langsci.2012.07.003	38	visual	violation	TAL	syntactically violation > correct sentence	-59	-33	38
							42	-52	58
							6	-65	45
							24	-33	-28
Koizumi et al. (2016)	10.3389/fpsyg.2016.01541	16	visual	complexity	MNI	complex Subject-Verb-Object > simple Verb-Object-Subject	-42	44	1
Kristensen et al. (2013)	10.1016/j.jneuroling.2012.05.001	21	visual	complexity	MNI	object-initial > subject-initial sentence	-6	10	58
							-54	14	10
							-38	-2	58
							10	8	2
							32	22	-2
							-52	-50	4
							-28	-60	46

							-14	-68	56
							-14	-10	6
							-10	10	4
Kristensen et al. (2014)	10.1162/jocn_a_00681	32	auditory	complexity	MNI	object-initial > subject-initial sentence in the main task	0	18	44
							-14	-74	-28
							32	28	-6
Kroczek et al. (2020)	10.1093/texcom/tgaa021	28	auditory	complexity	MNI	complex OSV > easy SOV sentence	-54	11	5
							-42	2	53
							-54	-40	2
Kunert et al. (2015)	10.1371/journal.pone.0141069	19	auditory	complexity	MNI	object-extracted relative clause > subject-extracted relative clause	-54	18	28
Kuperberg et al. (2000)	10.1162/089892900562138	9	auditory	violation	TAL	syntactically violated > normal sentence	-26	-14	-13
							12	-31	-2
							-17	-33	-2
							-38	-42	-2
							-35	-69	9
							-49	-53	9

							49	-14	-2
							-46	-47	15
							46	22	-2
							20	-53	4
							20	-53	-2
							-3	-78	-2
							-9	-53	4
							-14	-50	9
							14	-50	15
							9	-56	-7
							-12	-22	9
Kuperberg 2008	10.1016/j.neuroimage.2007.10.009	16	visual	violation	TAL	syntactic violated > normal sentence	-49	3	7
							29	19	5
							-45	-9	41
							52	3	33
							-19	33	29
							20	39	21
							-56	-31	38
							-51	-19	46
							-39	-4	14
							36	-3	14
							-53	-20	-1
							58	-19	3
							-6	37	-5

							6	49	-8
							-11	-6	47
							9	-2	52
							-35	-42	-6
							35	-38	-9
							-32	-92	17
							25	-80	36
							-12	11	-4
							28	-15	8
Lee and Newman (2010)	10.1002/hbm.20845	18	visual	complexity	MNI	Object-relative > co-joined active sentence in the sentence reading phase	-42	4	28
						Object-relative > co-joined active sentence in the probe phase	-46	30	-6
							46	15	34
							-38	2	60
							-32	20	-14
							-2	8	60
							-62	-34	2
							-28	-56	40

							38	-58	38
							0	-70	42
							8	-74	-30
							40	-64	-36
Lee et al. (2016)	10.1016/j.heares.2015.12.008	26	auditory	complexity	MNI	object-relative > subject relative	-60	-55	14
							-12	-13	8
							-48	17	20
							33	29	2
							18	-73	-28
							-39	-70	-34
							-9	-79	-28
							-3	17	53
							-6	38	44
							-9	20	32
							39	-1	50
							48	23	23
							45	5	38
							-60	-7	14
							-48	-13	20
							24	65	14
							42	56	2
							36	59	14
							63	-7	17
							66	-19	26

							63	-4	29
Lee 2018	10.1523/eneuro.0263-17.2018	35	auditory	complexity	MNI	Object-relative > subject-relative center-embedded clause	-48	-46	11
							-63	-46	8
							-54	-43	2
Lee 2023	10.1016/j.jneuroling.2023.101126	31	visual	complexity	MNI	topicalization > baseline sentence	-42	5	26
						object relative clause > baseline sentence	-51	29	2
							-57	-28	-2
							66	-13	-6
							63	-1	26
						subject-relative clause > baseline sentence	-45	-37	-2
							-39	2	42
							54	-28	-2
							-39	-52	-22
Makuuchi et al. (2013)	10.1093/cercor/bhs058	22	visual	complexity	MNI	syntactic movement distance	-36	6	33
							-51	15	18
							-33	-51	36
							9	-66	42
							-54	-36	-6
							-21	-15	6

							33	-45	39
							45	21	21
Matchin et al. (2014)	10.1016/j.bandl.2014.09.001	26	auditory	complexity	TAL	syntactically long-distance dependency > short-distance dependency	-50	16	25
Matchin 2016	10.3389/fpsyg.2016.00241	20	visual	complexity	TAL	Passive > active sentences	-32	-36	51
Meltzer et al. (2010)	10.1093/cercor/bhp249	24	auditory	complexity	TAL	object-embedded clause > subject-embedded clause	-45	12	13
Meyer 2012	10.1016/j.neuroimage.2012.05.052	24	auditory	complexity	MNI	object-first > subject-first sentence	-54	14	13
Nakagawa 2022	10.3389/fnhum.2021.753245	30	visual	complexity	MNI	Double Object > Prepositional Object structures	-46	50	8
							-48	42	16
							0	16	54
							4	22	46
							56	16	26
							-48	-36	42
							-44	-44	54

							-48	26	36
							-46	6	22
							-54	20	26
							-40	4	30
							-58	16	16
Newman et al. (2010)	10.1016/j.bandl.2010.02.001	20	visual	complexity	MNI	object-relative > co-joined sentence	-40	14	24
							-30	22	0
							-58	-36	2
Nieuwland et al. (2012)	10.1002/hbm.21377	20	visual	violation	MNI	case violation > correct	0	-30	28
							-8	0	36
							-4	-28	46
							-18	-68	32
							4	-70	40
							-4	-70	44
							50	-44	40
							60	-38	32
							46	-58	46
							-42	-46	42
							-52	-46	44
							-60	-34	36
						number agreement violation > correct	36	-52	42
							42	-44	38

							-44	32	34
							-44	54	8
							-44	48	24
							-42	-46	42
							-46	-36	36
							44	46	16
							50	40	20
							42	50	6
Obleser et al. (2011)	10.1016/j.neuroimage.2011.03.035	16	auditory	complexity	MNI	correlation with increasingly complex syntactic structure	-50	16	-20
							-64	-54	10
							-52	12	14
							44	12	18
							52	16	2
Obleser et al. (2011)	10.1016/j.neuroimage.2011.03.035	14	auditory	complexity	MNI	correlation with increasingly complex syntactic structure	-48	10	18
Ogawa et al. (2008)	10.1097/WNR.0b013e3282ffda89	21	visual	complexity	MNI	center-embedded > left-branching	-36	10	40
							-40	18	28
						center embedded > active-co-joined sentence	-34	8	44
							-28	-52	42

							32	-48	50
							30	6	50
							0	26	54
							10	-74	-36
							38	16	28
Ohta 2017	10.3389/fpsyg.2017.00748	17	visual & auditory	complexity	MNI	VSO > VOS	-36	6	57
							-48	0	45
							-48	12	42
							-57	12	12
							-54	24	0
							-6	15	48
							-12	-78	45
							-27	-69	39
							-30	-75	24
							18	-69	51
							30	-66	51
						OVS > SVO	-42	3	54
							-51	18	27
							-54	30	0
							-3	15	60
							-6	15	48
							-3	27	42
						VSO + OVS > VOS + SVO	-42	3	51
							-45	12	42

							-36	12	33
							-48	18	27
							-54	12	15
							-54	27	0
							-6	15	48
							0	24	42
							-27	-69	42
Pattamadilok 2016	10.1016/j.cortex.2015.11.012	20	visual	complexity	MNI	embedded > adjunct sentence during probe presentation	-48	20	28
							-54	20	19
							-48	29	22
							-57	20	1
							54	26	28
							-48	26	-8
							-48	38	-8
							-27	23	-5
							33	26	-5
							-57	-37	-2
							-51	-46	4
						embedded > adjunct sentence during related probe presentation	-27	23	-2
							-48	38	-8
							-54	23	19
							-51	17	10

							-51	14	40
							33	23	-5
							54	23	34
							-39	53	1
							0	32	49
							-3	14	55
							-3	5	67
							-39	2	43
							-57	-43	1
							-36	-55	46
							3	-64	46
Peelle 2004	10.1016/j.bandl.2004.05.007	8	auditory	complexity	TAL	Object-relative > Subject-relative center-embedded clause	-44	15	-7
							16	23	-8
Prat et al. (2011)	10.1093/cercor/bhq241	27	visual	complexity	MNI	Sentence with object-relative clause > co-joined clauses	-44	16	14
							-26	30	-2
							-40	4	48
							-6	10	64
							-12	58	24
							6	50	-16
							-58	-10	-20
							36	26	12
							54	30	0
							34	-2	44

							50	-10	-26
							50	-46	10
							26	-32	26
							-12	-12	-18
							22	-6	-14
							8	12	14
							8	-2	-8
							-30	-32	14
							-10	-60	-28
Quinones 2014	10.1016/j.neuroimage.2013.11.038	21	visual	violation	MNI	person mismatch > unagreement	2	34	34
							-44	22	36
							-40	46	22
							-32	46	34
							-42	-46	58
							-6	-66	50
							10	-32	52
							6	40	26
							56	-42	50
							58	-36	44
Quinones 2018	10.1016/j.neuroimage.2018.03.069	47	visual	violation	MNI	gender mismatch > match	-4	52	-2
							-26	24	50
							-6	38	-6
							-6	-22	60

							-42	-6	32
							-44	-16	34
							-48	-66	42
							-4	-48	10
							-8	-40	26
							-16	-82	28
							-4	-74	-2
							10	52	2
							26	54	6
							50	12	42
							34	-2	16
							14	14	12
							26	8	10
							2	-16	68
							8	-70	-4
Raettig 2010	10.1016/j.cortex.2009.06.003	15	auditory	violation	TAL	morphosyntactically incorrect > correct	-65	-42	15
Rogalsky 2008	10.3389/neuro.09.014.2008	15	visual	complexity	TAL	object-relative > subject-relative sentence	-41	38	14
							-42	13	23
							-56	-37	19
							-54	4	31
							-39	32	32
							-32	56	19

							47	16	20
Röder et al. (2002)	10.1006/nimg.2001.1026	11	auditory	complexity	TAL	syntactically difficult > easy (word order)	-45	12	16
							-47	-45	9
							-44	3	36
							-2	6	50
							31	19	2
Seyfried 2023	10.1080/23273798.2022.2116462	25	auditory	violation	MNI	Syntactic violation > correct	-32	28	-2
							-38	22	0
							-46	8	6
							-16	32	28
							-6	10	56
							-8	14	28
							14	-30	26
							-6	-30	24
							22	-34	30
Shetreet 2009	10.1016/j.neuroimage.2009.07.001	19	auditory	complexity	TAL	sentence with sentencial complement > sentence with noun phrase complement	-56	21	7
							-48	-35	5
							45	-24	-6
							-53	-15	-14
							50	2	-25
							-59	-51	27

							56	-48	22
							-9	-54	33
							-12	-25	29
							-18	-9	0
							-18	45	34
							-30	5	38
Shetreet 2014	10.1016/j.jneuroling.2013.06.003	22	auditory	complexity	TAL	wh-movement > canonical order	-53	24	10
							50	27	-5
							-53	-27	0
							51	-35	2
							-12	-81	-26
							-48	-1	50
						verb movement > canonical order	-18	-94	-7
Snijders 2009	10.1093/cercor/bhn187	22	visual	ambiguity	MNI	word-class ambiguous > unambiguous sentence	-52	-50	-8
							-60	-44	-4
							-46	-54	-4
							-50	-20	-28
							-42	-24	-28
							-46	-30	-22
							48	-34	-14
							50	-44	-4
							-44	0	22

							46	28	6
							44	18	14
							54	18	12
Stowe 2004	10.1016/S0093-934X(03)00359-6	16	visual	ambiguity	TAL	syntactically ambiguous > unambiguous sentences	-48	20	28
							18	6	16
							38	-80	-24
							-14	36	44
Stromswold 1996	10.1006/brln.1996.0024	8	visual	complexity	TAL	center-embedded > right-branching sentence	46.50	9.80	4.00
Suh et al. (2007)	10.1016/j.brainres.2006.12.043	16	visual	complexity	TAL	embedded > conjoined sentence	-38	-52	46
							-8	-70	46
							-46	16	46
Tanaka 2017	10.2183/pjab.93.031	16	visual	complexity	MNI	object scrambling > unscrambling sentence	-42	-1	26
							-48	14	20
							-60	11	17
Thibault 2021	10.1126/science.abe0874	20	visual	complexity	MNI	2 object-relative clauses > (coordinated clauses + subject-relative clauses	-18	14	-1
							-42	29	5
							-42	-52	32

							15	11	-1
							51	-49	26
Xiong 2021	10.1016/j.neuroimage.2020.117475	29	visual	complexity	MNI	center-embedding object relative clause > left-branching object relative clause	-42	-60	48
							-38	14	26
							46	-40	42
						center-embedding object relative clause > center-embedding subject-relative clause	-56	-8	-14
							-48	-58	22
Xu 2020	10.1016/j.bandl.2019.104712	19	visual	complexity	MNI	hard subject-extracted relative clause - visual baseline > easy object-extracted relative clause - visual baseline in Chinese	-34	28	-4
							-60	-50	16
Ye and Zhou (2009)	10.1016/j.neuroimage.2009.06.032	19	visual	complexity	MNI	Passive > active sentences	-12	8	60
							-54	22	14
							-32	30	-4
							34	-88	-10
Zhang 2024	10.1016/j.neuroimage.2024.120543	29	visual	complexity	MNI	marked non-canonical > canonical word order sentence	-54	-42	0
							-42	0	45
							-54	18	18
							-42	27	-9

							-3	15	51
						unmarked non-canonical with same-animacy arguments > canonical sentence	-54	15	18
							-39	3	39
							-54	15	54
							-54	18	6
							-51	33	-9
							-48	9	-9
							-36	-48	36
							-42	0	45
							-54	-48	0
							-63	-51	45
							-48	18	48
							30	-66	-54
							-30	3	-33
							42	3	33
						unmarked non-canonical with different animacy argument > canonical sentence	-54	-39	-3
							-36	-48	-3
							-48	-42	36
							-54	18	-9
							-51	30	-6
							-27	0	48

							-39	0	33
							-54	18	15
							-6	12	57

Note: TAL = talairach space.

Syntax violation and complexity

To assess whether this result was driven by a single type of task contrast or true across the different assessments of syntax, we compared the two main contrast types: syntactic complexity and violation (Supplementary Figure 1 and Table 2). The two studies on syntactic ambiguity were excluded for this step only. Assessments of syntactic complexity recruits a very similar network to the overall syntax finding, including left IFG (pars opercularis and pars triangularis) extending into precentral gyrus and MFG, pSTS/MTG, dmPFC, and IPL, as well as right IFG (pars orbitalis)/insula. This is not surprising as this includes the majority of the syntax studies (50 out of 66). In contrast, the syntactic violation network is quite limited due to it only including 14 studies. The two contrast types overlap in a small cluster in the left IFG (pars opercularis). While there is significantly greater involvement of a small area of left IFG (pars triangularis) for the complexity studies this could be due to the lack of power in the violation assessment.

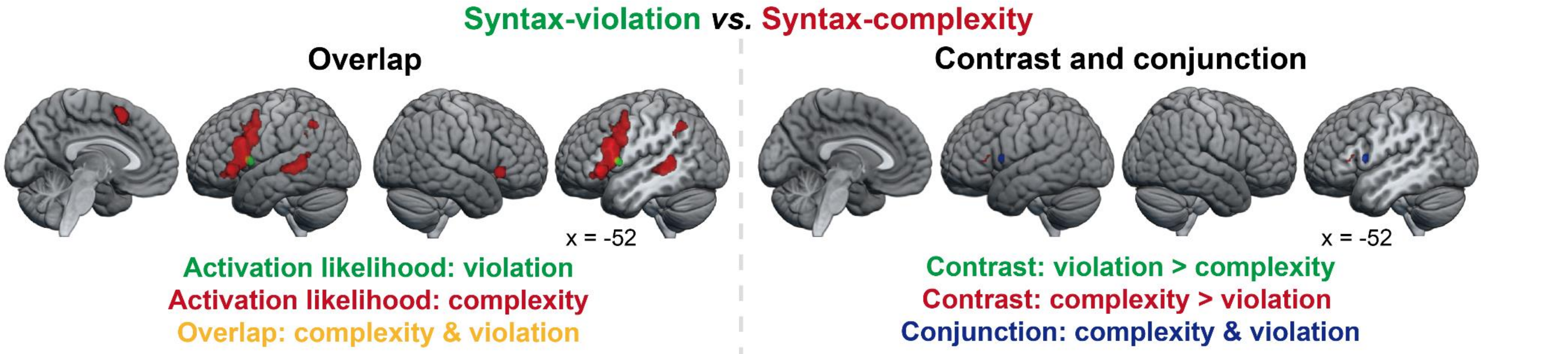

Supplementary Figure 1 Overlap of ALE results for violation and complexity (left, overlap in yellow). Contrast and conjunction analysis between violation and complexity at a voxel-level $p < 0.001$ with 10,000 permutations, cluster volume > 20 mm^3 right, auditory violation > complexity in green, complexity> violation in red, conjunction in blue).

Supplementary Table 2 Contrast analyses between syntax complexity and syntax violation

Cluster number	Region	Peak MNI Coordinates		
		X	Y	Z
Contrast analysis: Syntax complexity > syntax violation				
1	L IFG (pars triangularis)	-53	30	6
		-51	24	10
Contrast analysis: Syntax violation > syntax complexity				
	No significant results			
Conjunction analysis: syntax violation & syntax complexity				
1	L IFG (pars opercularis)	-50	10	8

Note: IFG = inferior frontal gyrus. Voxel-level $p < 0.001$ with 10,000 permutations, cluster volume > 20 mm^3.
